# Association between type of exercise and health-related quality of life in adults without activity limitations: a nationwide cross-sectional study

**DOI:** 10.1186/s12889-020-08699-1

**Published:** 2020-05-01

**Authors:** Min-Jung Choi, Yong Gyu Park, Yang Hyun Kim, Kyung Hwan Cho, Ga Eun Nam

**Affiliations:** 1 Department of Nursing, College of Nursing, The Catholic University of Korea, Seoul, Republic of Korea; 2 Department of Biostatistics, College of Medicine, The Catholic University of Korea, Seoul, Republic of Korea; 3 Department of Family Medicine, Korea University Anam Hospital, Korea University College of Medicine, Seoul, Republic of Korea

**Keywords:** Quality of life, EQ-5D, EQ-VAS, Walking, Flexibility exercises, Resistance training

## Abstract

**Background:**

Exercise is known to be associated with health-related quality of life (HRQoL), however, evidence on the association between type of exercise and HRQoL in the general population is limited. We performed this study to investigate the association of exercise types and their combinations with HRQoL in Korean adults.

**Methods:**

We analyzed data from 13,437 adults aged ≥19 years without activity limitations who had participated in the 5th Korea National Health and Nutrition Examination Survey 2010–2012. As per the American College of Sports Medicine guideline, exercise types were categorized into eight groups: walking (W), flexibility (F), resistance (R), W + F, W + R, F + R, and W + F + R exercise groups and a non-exercise group. The European Quality of Life-5 Dimension (EQ-5D) index and the European Quality of Life Visual Analogue Scale (EQ-VAS) were used to assess HRQoL.

**Results:**

The mean age of participants was 42.8 ± 0.2 years. The proportion of participants in the non-exercise group was the highest (34.7%); among the exercise groups, the walking group was the most prevalent (16.9%) and the W + R group was the least (1.2%). In analysis of covariance, the mean EQ-5D index in W (0.875), W + F (0.878), F + R (0.877), and W + F + R (0.876) groups was significantly higher compared with that in non-exerciser group (0.869) (*p* < 0.05). The mean EQ-VAS score in the W (64.064), F (64.427), W + F (65.676), F + R (65.811), and W + F + R (67.110) groups was higher than that in the non-exercise group (62.396) (*p* < 0.05). No difference was observed between R and W + R groups and non-exercise group with regard to the EQ-5D index and EQ-VAS score.

**Conclusions:**

The W (for 30 min at least five times a week), W + F, F + R (at least two days a week), and W + F + R groups showed higher HRQoL than the non-exercise group. This study may be helpful in the development of public exercise interventions, which could help enhance HRQoL in adults.

## Background

Regular physical activity (PA) is associated with a reduced risk of cardiovascular disease [1], diabetes [2], disability, depression, anxiety [3], and all-cause mortality [4]. Exercise is a subcategory of PA that is planned, structured, repetitive, and aims to improve or maintain one or more components of physical fitness [5]. The American College of Sports Medicine (ACSM) guidelines for PA categorize the exercise according to: types (including walking, flexibility, and resistance exercise), intensity (including vigorous to moderate exercise), frequency (day/week), and duration (minutes) [6]. Concerning types of exercise the ACSM recommends aerobic, flexibility, and resistance exercise [6]. Despite interventions to promote PA including exercise in the Korean general population, the Korea Centers for Disease Control and Prevention (KCDC) reported that walking exercise has decreased by 20.1% over the last decade, and aerobic and muscle strength exercise has reduced by 7.9% over the last three years [7].

Health-related quality of life (HRQoL) is defined as an individual’s perceived physical and mental health over time [8, 9]. As major health concerns have changed from communicable diseases to non-communicable diseases such as cardiovascular disease and cancer [10], interest in qualitative indicators such as HRQoL has increased. HRQoL is an important indicator for evaluating the effects of public health programs as well as monitoring mortality [11–13]. There are various tools to assess HRQoL. Among them, the European Quality of Life-5 Dimension (EQ-5D) questionnaire has been widely used to measure HRQoL in many population-based surveys because of its simplicity and brevity [14].

PA including exercise is associated with HRQoL [15–17]. Previous studies have examined the association between PA intensity—based on the International Physical Activity Questionnaire (IPAQ) [18]—and HRQoL. Cross-sectional studies with 5537 adults (40–60 years) in England [16], 2853 Korean elderly people [19], and 10,392 adults aged 40 years or over in Korea [20] have shown that higher levels of PA are associated with better HRQoL.; However, studies on the association between exercise type and HRQoL have been limited to patients with specific illnesses or older people [21, 22]. Previous studies have analyzed only three types of exercise—walking, flexibility, and resistance exercise—and have not considered the fact that individuals may perform two or more combined exercises. Moreover, exercise type is associated with a combination of individual characteristics such as age, sex, marital status, educational level, body mass index (BMI), disease, and circumstances such as infrastructure in the communities for PA [23]. There is limited evidence on the association between type of exercise and HRQoL considering various confounding factors including participants’ sociodemographics, health behaviors, and health conditions in the general population. The dearth of evidence is mainly because a comparison between seven exercise types (three types of exercise and combined exercises) and a non-exercise group requires a large sample and significant funding.

Therefore, to fill this gap in existing research, this study aimed to investigate the association between three types of exercise and combination exercises and HRQoL in adults, considering societal, environmental, and behavioral factors using nationwide data representative of the South Korean population.

## Methods

### Survey overview and study population

This population-based cross-sectional study used data obtained from the 5th Korea National Health and Nutrition Examination Survey (KNHANES V) performed between 2010 and 2012. The KNHANES is a cross-sectional and nationally representative survey to evaluate health and nutritional status and to calculate the health indices of the South Korean population. It has been conducted every year by the KCDC since 1998. This survey uses a stratified cluster sampling design for the selection of participants and consists of three parts: a health interview, a nutrition survey, and a health examination. The health interview of the KNHANES V was conducted at mobile examination centers, and the response rate was 80.0%. The KNHANES data are publicly available, and any researcher can download the data from the following website: https://knhanes.cdc.go.kr/knhanes/eng/index.do.

**Fig. 1:**
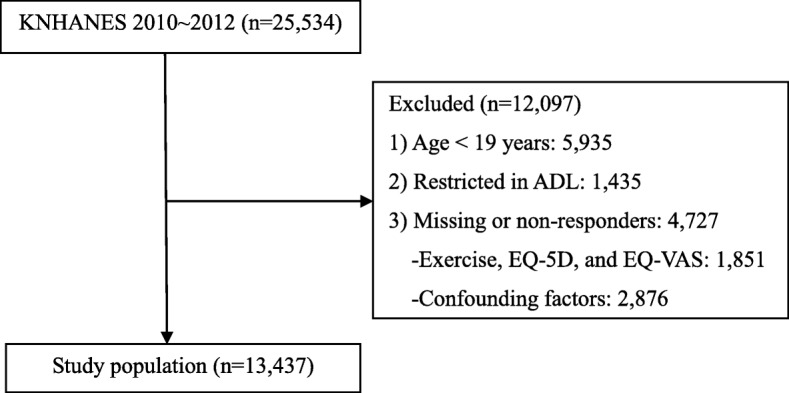
Participant flow diagram illustrating the number of excluded individuals

The KNHANES V included 25,534 participants. Of these, 19,599 participants were aged 19 years or older. The 4727 individuals who did not respond to the questions regarding exercise, HRQoL, or socioeconomic status were excluded from the present analysis. Further, 1435 respondents who reported being restricted in activities of daily living (ADL) and social activities owing to health problems or physical/mental disabilities were also excluded. Finally, a total of 13,437 participants were included in this study (Fig. 1). The research ethics committees of the KCDC approved the survey protocol, and informed consent was provided by all participants. This study was approved by the institutional review board of the Catholic University of Korea, St. Mary’s Hospital in Seoul (approval number: KC18ZESI0062).

### Assessment of type of exercise

Participants responded to the questionnaires regarding type and frequency of exercise. Regarding walking exercise, it was assessed using the Korean version of the IPAQ [18]. Participants were asked the following questions: “how many days did you walk for at least 10 minutes during the last 7 days?” and “how long did you walk per day on these days?” Regarding flexibility and resistance exercise, participants were asked the following: “how many days did you perform flexibility exercise such as stretches or free gymnastics focused on flexibility during the last 7 days?” and “during the last 7 days, how many days did you perform resistance exercise such as push-ups, sit-ups, dumbbells, weights, and chin-ups?” According to the guidelines recommended by the ACSM, the walking exercise group was defined as individuals who walked more than 30 min a day for more than five days a week. The flexibility and resistance exercise groups were defined as individuals who performed flexibility and resistance exercise for more than two days a week, respectively [6]. Subsequently, participants were classified into eight groups: a non-exercise group who did not perform any exercise as recommended, three groups performing only one type of exercise (walking [W], flexibility [F], and resistance [R] exercise), three groups performing two types of exercise together (walking and flexibility [W + F], walking and resistance [W + R], and flexibility and resistance [F + R]), and one group performing all three types of exercise (W + F + R).

### Assessment of HRQoL

HRQoL was evaluated using the Korean version of the European Quality of Life (EuroQoL) Scale [14], which consists of two parts: the health-status descriptive system EQ-5D and the EuroQoL Visual Analogue Scale (EQ-VAS). The EQ-5D 3 level comprises five dimensions (mobility, self-care, usual activities, pain/discomfort, and anxiety/depression), and each dimension is assessed based on a single question with three response levels (no problem, some problems, and extreme problems). Participants indicate their current health (on the day of the questionnaire administration) by ticking one of the three response boxes for each of the five dimensions. Combining these answers constitutes 243 health states EQ-5D scores were calculated using the weighted model to transform these health states into Korean population-based health states [24]. Scores in the Korean EQ-5D index range from − 1 to 1, where 0 ≤ a health status worse than death, 0 = death, and 1 = perfect health status [25]. Respondents also indicated their current subjective health status using the EQ-VAS, which is designed on a vertical ruler from 0 (worst imaginable health state) to 100 (best imaginable health state), in increments of one. The Korean versions of the EQ-5D and EQ-VAS have been evaluated for their validity and reliability [24, 26].

### Covariates

We considered age, sex, household income, education level, marital status, and area of residence as socio-demographic variables. Participants were divided according to age into: young-aged (19–39 years), middle-aged (40–64 years), and elderly (over 65 years) individuals. Household income was the total income of a household in the last year, including all income such as wages, real estate income, pensions, interest, and government subsidies. Household income was calculated as the equivalised income $\left(=\mathrm{total}\ \mathrm{household}\ \mathrm{income}\ \mathrm{per}\ \mathrm{month}/\sqrt{\mathrm{number}\ \mathrm{of}\ \mathrm{family}\ \mathrm{members}}\right)$. The quartiles were divided into lowest, lower intermediate, higher intermediate, and highest groups according to the sample household income quartile threshold in the KNHANES. Education level was divided into four groups (elementary school or below, middle school, high school, and university or higher). Marital status was reported as unmarried, with spouse, and without spouse. Regarding area of residence, participants dwelling in a “dong” (neighborhood) were defined as urban dwellers, whereas those living in an “eup” (town) or “myeon” (township) were defined as rural dwellers.

BMI was calculated as the individual’s body weight (kg) divided by the square of height (m), and then classified into three groups: underweight (< 18.5 kg/m^2^), normal to overweight (18.5–24.9 kg/m^2^), and obese (≥ 25 kg/m^2^) [27]. Participants were classified as never smokers, former smokers (had smoked ≥100 cigarettes during their lifetime but were not smoking currently), and current smokers (had smoked ≥100 cigarettes and were still smoking). Individuals with 8 points or higher on the Alcohol Use Disorder Identification Test (AUDIT) were considered problem drinkers [28]. ADL level was divided into three groups (light, normal, and severe activity). Participants were also asked whether they had ever been diagnosed by a physician with hypertension, diabetes, stroke, heart diseases (myocardial infarction or angina), arthritis (osteoarthritis or rheumatoid arthritis), or any malignancy.

### Statistical analysis

The SAS survey procedure (version 9.3, SAS Institute, Cary, NC, USA) was used to account for the complex sampling design. The KNHANES V is based on a two-stage stratified cluster sampling method with districts and households, and the sampling weights were obtained from these strata. Data were presented as mean ± standard error (SE) for continuous variables or as percentage (SE) for categorical variables. Participants’ characteristics according to type of exercise group were compared using one-way analyses of variance for continuous variables and Pearson chi-squared test for categorical variables. We compared the HRQoL indices (EQ-5D index and EQ-VAS) among eight exercise groups and calculated the significance of the difference using the analysis of covariance (ANCOVA) after adjustment for covariates. We then analyzed whether types of exercise are statistically different each other using Tukey’s test for post hoc analysis. Model 1 was adjusted for age and sex, whereas model 2 was adjusted for age, sex, household income, education level, marital status, and area of residence. Model 3 was additionally adjusted for BMI, smoking status, alcohol consumption, ADL, and past medical history based on previous studies [29, 30]. Associations between type of exercise and HRQoL indices according to age and sex were examined using an ANCOVA after adjusting for all potential confounding variables (age, sex, household income, education level, marital status, area of residence, BMI, smoking status, alcohol consumption, ADL, and past medical history). Statistical significance was defined by a two-tailed *p*-value < 0.05.

## Results

### Characteristics according to type of exercise

Participants’ socio-demographic and clinical characteristics according to exercise type groups are shown in Tables 1 and 2. The demographic, economic, and lifestyle characteristics were significantly different among the exercise groups (*p* < 0.001). Their mean age was 42.8 ± 0.2 years, with the highest among those in the F + R group (44.8 ± 0.5 years). The proportion of males was the highest in the W + R group (89.1%). Income and educational levels were significantly different among exercise groups (*p* < 0.001): the W group showed the highest rate of the lowest income level (lowest quartile group) and non-exerciser group showed the highest rate of the lowest educational level (≤elementary school). The proportion of urban dwellers was the highest in the W + F group. The proportions of unmarried respondents, current smokers, and problem drinkers were high among individuals in the R and W + R groups. The rate of underweight and obese individuals was the highest in the non-exercise group (6.3%) and the W + R group (38.2%), respectively. The prevalence of hypertension (*p* = 0.015) and arthritis (*p* < 0.001) significantly differed among the exercise groups.

**Table 1:** Socio-demographic characteristics of study participants according to type of exercise

	Total	Non-exerciser	Walking	Flexibility	Resistance	Walking + Flexibility	Walking +Resistance	Flexibility +Resistance	Walking +Flexibility +Resistance	P^*^
N	13,437	4659	2270	2058	227	1508	159	1249	1307	
Age (years)	42.8 ± 0.2	43.7 ± 0.3	41.2 ± 0.4	44.2 ± 0.4	39.6 ± 1.2	41.8 ± 0.4	36.1 ± 1.3	44.8 ± 0.5	41.8 ± 0.6	< 0.001
Sex (males)	53.3 (0.5)	49.6 (0.8)	52.9 (1.3)	40.8 (1.4)	84.3 (2.5)	40.5 (1.6)	89.1 (2.6)	68.2 (1.5)	71.8 (1.6)	< 0.001
Household income										< 0.001
Lowest	12.3 (0.5)	13.3 (0.7)	14.3 (0.9)	10.3 (0.8)	9.1 (1.9)	12.6 (1.1)	13.1 (3.1)	9.5 (1.0)	10.9 (1.3)	
Lower intermediate	27.0 (0.7)	28.0 (1.0)	28.8 (1.2)	26.2 (1.4)	32.5 (4.6)	26.0 (1.5)	27.1 (4.6)	23.2 (1.8)	25.2 (1.7)	
Higher intermediate	31.0 (0.7)	31.3 (1.0)	30.7 (1.3)	31.8 (1.3)	29.4 (4.3)	31.5 (1.6)	30.2 (4.2)	29.5 (1.8)	30.6 (1.5)	
Highest	29.7 (0.8)	27.4 (1.1)	26.2 (1.3)	31.7 (1.5)	28.9 (4.1)	29.9 (1.7)	29.7 (4.6)	37.8 (1.9)	33.4 (1.8)	
Education level										< 0.001
≤ Elementary school	13.2 (0.5)	17.3 (0.8)	15.7 (1.0)	12.7 (0.8)	12.6 (2.2)	10.0 (0.8)	8.3 (2.3)	8.0 (1.0)	5.8 (0.7)	
Middle school	9.4 (0.3)	10.1 (0.6)	8.3 (0.7)	9.2 (1.0)	7.6 (2.3)	8.0 (0.8)	8.6 (2.8)	11.1 (1.1)	9.5 (1.0)	
High school	42.1 (0.6)	38.2 (1.0)	43.7 (1.4)	39.5 (1.5)	53.6 (4.2)	46.2 (1.7)	49.0 (4.8)	43.5 (1.8)	47.4 (1.8)	
≥ University	35.3 (0.7)	34.5 (1.1)	32.3 (1.2)	38.7 (1.5)	26.3 (3.5)	35.9 (1.6)	34.2 (4.6)	37.5 (1.9)	37.4 (1.7)	
Marital status										< 0.001
With spouse	68.0 (0.7)	72.7 (0.9)	60.6 (1.5)	74.8 (1.3)	50.0 (4.0)	63.8 (1.5)	37.0 (4.6)	73.9 (1.8)	62.5 (1.9)	
Without spouse	7.0 (0.3)	7.9 (0.5)	6.7 (0.6)	7.4 (0.7)	6.5 (1.8)	7.4 (0.8)	6.5 (2.4)	6.6 (0.8)	4.7 (0.7)	
Unmarried	25.0 (0.7)	19.4 (0.9)	32.7 (1.5)	17.8 (1.2)	43.6 (4.2)	28.9 (1.6)	56.5 (4.9)	19.5 (1.7)	32.8 (1.9)	
Area of residence (Urban)	82.0 (1.6)	78.2 (2.0)	80.7 (1.9)	82.8 (1.9)	77.4 (5.0)	87.4 (1.8)	84.0 (3.7)	86.5 (1.7)	86.7 (1.7)	< 0.001

Data are presented as mean ± standard error (SE) or percentage (SE)

^*^P-values were obtained using a one-way analysis of variance for continuous variables and a Pearson chi-squared test for categorical variables

**Table 2:** Clinical characteristics of study participants according to type of exercise

	Total	Non-exerciser	Walking	Flexibility	Resistance	Walking + Flexibility	Walking +Resistance	Flexibility +Resistance	Walking +Flexibility +Resistance	P^*^
N	13,437	4659	2270	2058	227	1508	159	1249	1307	
Smoking status										< 0.001
Current smoker	29.2 (0.6)	30.3 (0.8)	31.4 (1.3)	23.0 (1.2)	47.5 (4.5)	23.8 (1.5)	37.0 (4.7)	29.8 (1.7)	31.4 (1.8)	
Former smoker	21.0 (0.4)	18.9 (0.7)	18.5 (1.0)	18.9 (1.1)	19.7 (2.8)	17.5 (1.2)	28.5 (4.3)	30.4 (1.6)	29.2 (1.5)	
Non-smoker	49.8 (0.5)	50.8 (0.8)	50.1 (1.4)	58.1 (1.4)	32.8 (4.1)	58.7 (1.6)	34.5 (4.7)	39.8 (1.8)	39.5 (1.7)	
Problem drinker	38.4 (0.5)	36.6 (0.8)	39.3 (1.4)	32.6 (1.3)	56.3 (4.4)	32.7 (1.7)	52.5 (4.9)	42.3 (1.8)	47.9 (1.8)	< 0.001
Activities of daily living										< 0.001
Light	51.4 (0.6)	56.7 (0.9)	48.3 (1.4)	53.9 (1.4)	58.9 (3.8)	45.2 (1.6)	49.9 (4.8)	47.0 (1.8)	45.2 (1.7)	
Normal	39.1 (0.5)	35.3 (0.8)	40.3 (1.3)	40.9 (1.3)	30.8 (3.7)	45.2 (1.6)	36.9 (4.8)	42.5 (1.8)	39.6 (1.8)	
Severe	9.5 (0.4)	8.1 (0.6)	11.5 (1.0)	5.3 (0.6)	10.4 (2.8)	9.5 (1.1)	13.3 (3.2)	10.6 (1.2)	15.3 (1.4)	
BMI (kg/m^2^)										< 0.001
< 18.5	4.8 (0.2)	6.3 (0.5)	6.2 (0.7)	3.9 (0.5)	2.6 (1.2)	4.4 (0.6)	2.6 (1.4)	2.0 (0.5)	2.9 (0.6)	
18.5–25	63.6 (0.5)	61.2 (0.9)	67.3 (1.2)	64.4 (1.4)	65.6 (4.3)	64.3 (1.5)	59.2 (4.8)	63.6 (1.8)	64.0 (1.7)	
≥ 25	31.5 (0.5)	32.5 (0.9)	26.5 (1.2)	31.6 (1.3)	31.8 (4.1)	31.3 (1.5)	38.2 (4.8)	34.4 (1.9)	33.1 (1.7)	
Past medical history										
Hypertension	13.4 (0.4)	15.0 (0.7)	11.7 (0.7)	13.1 (0.8)	12.9 (2.3)	11.9 (1.0)	12.0 (2.5)	14.5 (1.2)	12.6 (1.0)	0.015
Diabetes mellitus	4.8 (0.2)	5.5 (0.4)	4.2 (0.5)	3.7 (0.4)	4.3 (1.5)	4.5 (0.6)	6.5 (2.1)	4.9 (0.7)	5.2 (0.7)	0.145
Malignancy	1.0 (0.1)	0.9 (0.2)	0.9 (0.2)	1.4 (0.3)	0.9 (0.5)	0.9 (0.2)	0.3 (0.3)	0.7 (0.2)	1.0 (0.2)	0.416
Stroke	0.5 (0.1)	0.5 (0.1)	0.4 (0.1)	0.4 (0.1)	0.4 (0.3)	0.5 (0.2)	0.4 (0.3)	0.5 (0.2)	0.5 (0.3)	0.998
CVD	1.3 (0.1)	1.2 (0.2)	1.4 (0.3)	1.4 (0.3)	1.0 (0.7)	1.0 (0.2)	0.7 (0.5)	1.4 (0.3)	1.3 (0.3)	0.896
Arthritis	6.0 (0.2)	6.8 (0.4)	6.5 (0.6)	7.2 (0.6)	4.1 (1.6)	6.0 (0.7)	2.5 (1.3)	4.4 (0.6)	3.7 (0.5)	< 0.001

*Abbreviations: BMI* body mass index, *CVD* cardiovascular disease

Data are presented as percentage (SE)

^*^P-values were obtained using a Pearson chi-squared test

### Analysis of covariance regarding associations between type of exercise and HRQoL

Table 3 presents the results from the analysis of covariance between type of exercise and HRQoL indices. The EQ-5D index and EQ-VAS scores were different among the eight groups after adjusting for confounding variables (*p* < 0.001). In model 1, the W + F, F + R, and W + F + R groups exhibited higher scores of the EQ-5D index compared with the non-exercise group (*p* < 0.05). EQ-VAS scores in the F, W + F, W + F, W + F + R groups were significantly higher than the non-exercise group (*p* < 0.05). These associations persisted after further adjustment for confounding variables in models 2 and 3. In model 3, EQ-5D index in W (0.875 ± 0.013), W + F (0.878 ± 0.013), F + R (0.877 ± 0.013), and W + F + R (0.876 ± 0.013) groups was significantly higher than that in the non-exercise group (0.869 ± 0.013) (*p* < 0.05). EQ-VAS score in W (64.064 ± 1.549), F (64.427 ± 1.530), W + F (65.676 ± 1.491), F + R (65.811 ± 1.565), and W + F + R (67.110 ± 1.585) groups was also significantly higher than that in the non-exercise group (62.396 ± 1.530) (*p* < 0.05).

**Table 3:** Analysis of covariance of HRQoL indices according to type of exercise

	EQ-5D index		EQ-VAS	
	Adjusted Mean ± SE	*P*	Adjusted Mean ± SE	*P*
Model 1				
W + F + R	0.966 ± 0.003	< 0.001	78.096 ± 0.489	< 0.001
F + R	0.970 ± 0.003		77.195 ± 0.497	
W + R	0.961 ± 0.005		75.266 ± 1.405	
W + F	0.968 ± 0.003		76.541 ± 0.447	
R	0.952 ± 0.005 ^F + R^		75.770 ± 1.273	
F	0.962 ± 0.003 ^F + R^		75.581 ± 0.385 ^W + F + R^	
W	0.961 ± 0.003 ^W + F, F + R^		74.490 ± 0.431 ^W + F, F + R, W + F + R^	
Non	0.956 ± 0.002 ^W + F, F + R, W + F + R,^		73.070 ± 0.338 ^F, W + F, F + R, W + F + R^	
Model 2				
W + F + R	0.954 ± 0.003	< 0.001	77.188 ± 0.582	< 0.001
F + R	0.955 ± 0.003		75.964 ± 0.566	
W + R	0.955 ± 0.006		75.142 ± 1.417	
W + F	0.956 ± 0.003		75.673 ± 0.524	
R	0.944 ± 0.005		75.269 ± 1.272	
F	0.949 ± 0.003		74.362 ± 0.476 ^W + F + R^	
W	0.952 ± 0.003		73.899 ± 0.522 ^F + R, W + F + R^	
Non	0.945 ± 0.002 ^W, W + F, F + R, W + F + R^		72.151 ± 0.428 ^W, F, W + F, F + R, W + F + R^	
Model 3				
W + F + R	0.876 ± 0.013	< 0.001	67.110 ± 1.585	< 0.001
F + R	0.877 ± 0.013		65.811 ± 1.565	
W + R	0.878 ± 0.014		64.911 ± 2.113	
W + F	0.878 ± 0.013		65.676 ± 1.491	
R	0.868 ± 0.014		65.523 ± 1.957	
F	0.872 ± 0.013		64.427 ± 1.530 ^W + F + R^	
W	0.875 ± 0.013		64.064 ± 1.549 ^W + F + R^	
Non	0.869 ± 0.013 ^W, W + F, F + R, W + F + R^		62.396 ± 1.530 ^W, F, W + F, F + R, W + F + R^	

*Abbreviations: HRQoL* health-related quality of life, *EQ-5D* EuroQol 5-dimension, *EQ-VAS* EuroQol visual analogue scale, *Non* non-exerciser, *W* walking, *F* flexibility, *R* resistance, *SE* standard error,

*P*-values were obtained by ANCOVA

Superscripts indicate statistically different groups (*p* < 0.05) with the corresponding type of exercise by Tukey’s test for post hoc analysis

Model 1 was adjusted for age and sex

Model 2 was adjusted for model 1 plus household income, education level, marital status, and area of residence

Model 3 was adjusted for model 2 plus smoking status, alcohol drinking, activity of daily living, body mass index, and past medical history

### Adjusted means of HRQoL indices according to sex, age, and type of exercise

**Fig. 2:**
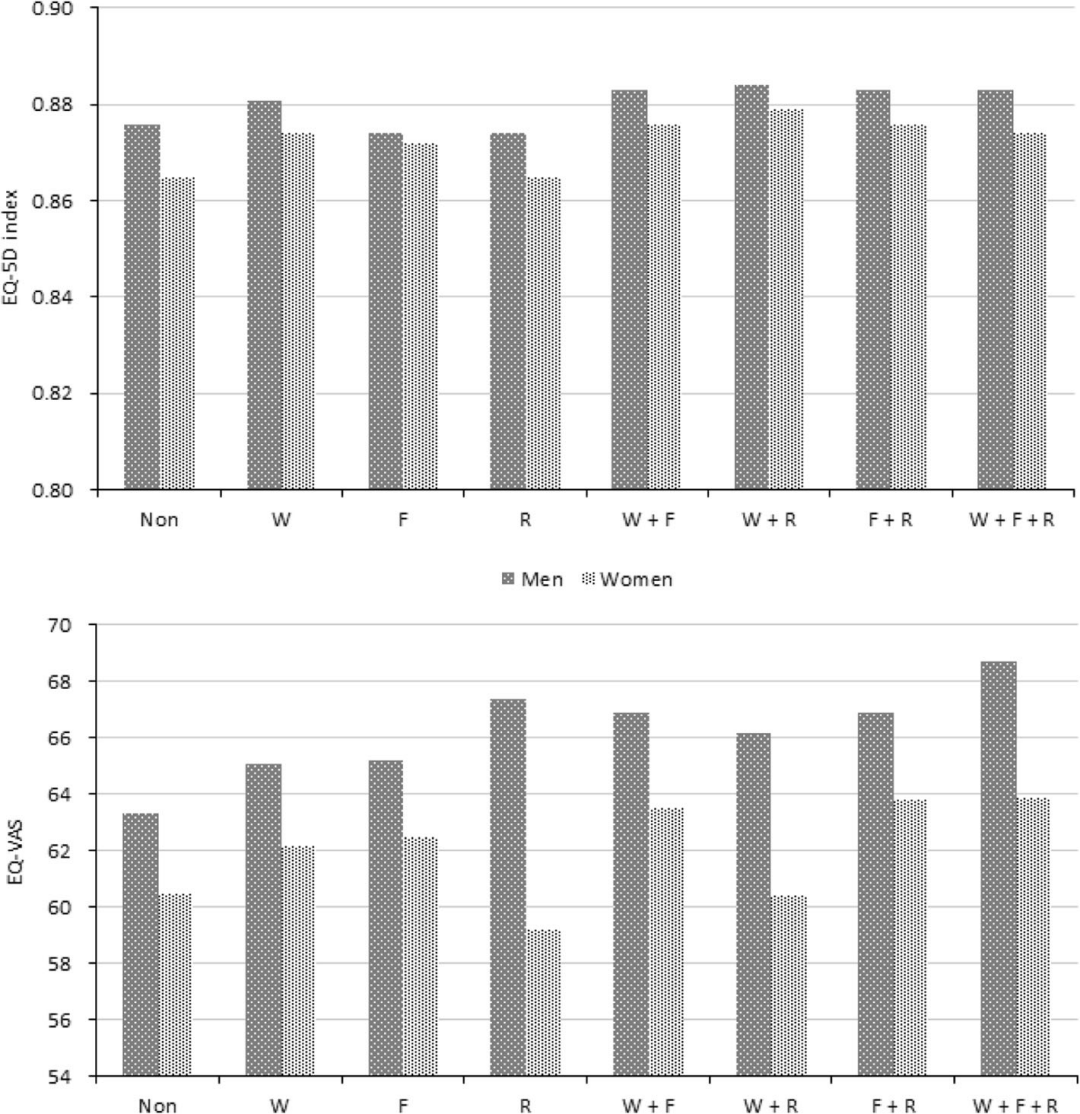
Adjusted means of EQ-5D index and EQ-VAS score according to sex and type of exercise

**Fig. 3:**
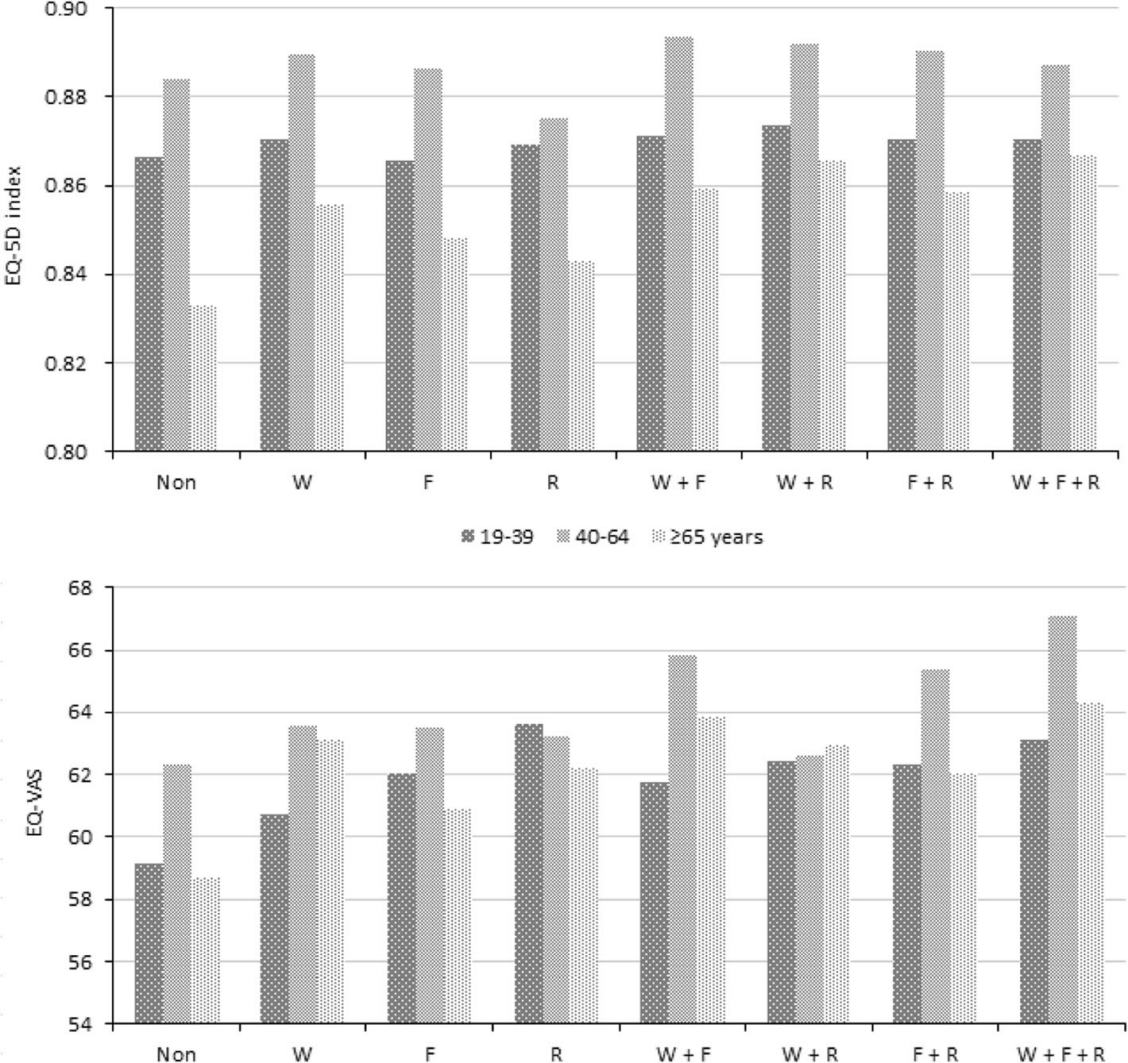
Adjusted means of EQ-5D index and EQ-VAS score according to age and type of exercise

Figures 2 and 3 show the adjusted means of the EQ-5D index and EQ-VAS score according to subgroups based on sex, age, and type of exercise after adjusting for all confounding variables. Both indices were higher in men than in women in all exercise type groups (Fig. 2). As for age, the mean of EQ-5D index was the highest in the middle-aged group (40–64 years), followed by the younger (19–39 years) and older (≥65 years) age groups (Fig. 3). Regarding EQ-VAS score, a similar pattern was shown in the non-exerciser, F, and F + R groups.

## Discussion

This study examined the association between type of exercise and HRQoL using a nationally representative data of South Korean adults. Compared with the non-exercise group, the EQ-5D index was significantly higher in the W, W + F, F + R, and W + F + R exercise groups, and the EQ-VAS score was higher in the W, F, W + F, F + R, and W + F + R exercise groups. These findings indicate that walking exercise and combinations of two or three types of exercise as recommended may positively affect HRQoL.

Previous studies on HRQoL have identified age, sex, education, income, BMI, stress, and the number of chronic diseases as factors affecting EQ-5D index [29, 31, 32]. The present study considered these confounding variables in the analysis of the association between types of exercise and HRQoL. With respect to these variables, we found that the proportion of participants performing the recommended exercises was lower among individuals with lower socioeconomic status, without a spouse, with a rural residence, with low BMI (underweight), women, and older ages (Table 1).

A key question in this study was whether type of exercise is associated with HRQoL compared to individuals who do not exercise. Based on the results using two HRQoL assessment tools, walking and combined exercise types showed significant positive associations with HRQoL indices. Walking is positively correlated with the EQ-5D index, which is consistent with findings from a previous questionnaire survey of 351 healthy adults [33] and another study that showed increased HRQoL in adults after walking exercise for 12 weeks [34]. Further, a combination of aerobic and flexibility exercise such as Tai chi and Qigong [35] and resistance and flexibility exercise [36] improved HRQoL, which is similar to our findings. The EQ-VAS score was higher in the W + F + R group than the non-exercise, W, and F groups. This finding is consistent with a previous study in which the HRQoL of participants who performed three combined exercise types was improved [37]. These results indicate that it may be necessary to consider combined exercise types in the development of public exercise interventions to improve HRQoL.

We found no differences in the EQ-5D index between groups that performed only flexibility or resistance exercise and the non-exercise group. A study for adults with metabolic syndrome reported that the EQ-5D index is not associated with flexibility or resistance exercise [22]. In elderly persons aged over 65 years, flexibility exercise was related to mobility and self-care, whereas resistance exercise was related only to mobility [21]. Thus, the association of flexibility and resistance exercise with HRQoL may be attributable to the characteristics of participants. Our results show that performing only flexibility or resistance exercise may not be sufficient for having a positive effect on the EQ-5D sub-dimensions. In addition, individuals performing only flexibility or resistance exercise may be doing so owing to limited flexibility or muscularity; in other words, they may not be in a state to perform other types of exercise, such as walking, or combined types of exercise.

Although the W + R group and the non-exercise group showed no significant difference in the EQ-5D index and EQ-VAS scores, HRQoL has been reported to improve with the performance of the W + R exercise in the general population as well as in those with heart failure and diabetes [38–41]. In the present study, a small number of participants performed only resistance exercise, and the sample of the W + R group was the smallest. Further research with larger samples is needed to reveal the association between performing W + R exercise and HRQoL. Moreover, as the number of participants who performed resistance exercise was too small, it is necessary to emphasize resistance exercise along with other types of exercise in public exercise interventions.

The EQ-VAS score was higher in the group that performed walking and flexibility exercise compared with the non-exercise group. This finding can be attributed to differences in the scoring mechanisms of the EQ-5D index and EQ-VAS. The EQ-5D index reflects general health status and includes a wide range of health problems by sub-dimension. The EQ-VAS, meanwhile, measures respondents’ health status on the day they complete the questionnaire and may not necessarily be associated with their current health status [42]. Internal factors, such as the satisfaction of performing any exercise regularly, are highly likely to affect the EQ-VAS score.

Given the overall design of this study, there are several limitations. First, there may be potential recall bias because the type of exercise and other lifestyle factors were based on information collected retrospectively using self-reported questionnaires. Second, we cannot confirm a causal association between type of exercise and HRQoL due to the cross-sectional design of our study. Third, this study did not measure the intensity of each exercise type that could influence HRQoL and this should be addressed in further studies. Fourth, there might be other activities (e.g. cycling) that participants could be involved and were not gathered in the established groups and might have influenced the participants’ HRQoL. Fifth, individuals with limitations of ADL or social activity due to health problems and physical or mental disabilities were excluded from the current analysis; therefore, it is necessary to conduct further studies by considering such individuals. Sixth, because this study was based on the nationwide data of South Korea, it cannot be generalized to other populations.

## Conclusions

This study showed that different types of exercise are differently associated with HRQoL in Korean adults. Walking and a combination of two or three types of exercises were positively associated with HRQoL. Our results may be helpful in the development of public exercise interventions to improve HRQoL in adults. Future research should examine the association of the combination of walking and resistance exercise with HRQoL. Moreover, it will be meaningful to consider a specific population, such as non-communicable diseases, in this regard. Further studies should be to perform a longitudinal study rather than cross-sectional, to observe long- term changes.

## Data Availability

Survey data supporting the conclusions of this article are publicly available at https://knhanes.cdc.go.kr/knhanes/main.do through the submission of a written plan for data utilization.

## Abbreviations

HRQoL: Health-related quality of life
KNHANES: Korea National Health and Nutrition Examination Survey
W: Walking
F: Flexibility
R: Resistance
EQ-5D: European Quality of Life-5 Dimension
EQ-VAS: European Quality of Life Visual Analogue Scale
ACSM: American College of Sports Medicine
KCDC: Korea Centers for Disease Control and Prevention
IPAQ: International Physical Activity Questionnaire
ADL: Activities of daily living
EuroQoL: European Quality of Life
BMI: Body mass index
AUDIT: Alcohol Use Disorder Identification Test
ANOVA: Analysis of Covariance
SE: Standard error
Non: Non-exerciser
